# A multi-disciplinary view on earthquake science

**DOI:** 10.1038/s41467-022-34955-6

**Published:** 2022-12-09

## Abstract

Earthquakes are a natural hazard affecting millions of people globally every year. Researchers are working on understanding the mechanisms of earthquakes and how we can predict them from various angles, such as experimental work, theoretical modeling, and machine learning. We invited Marie Violay (EPFL Lausanne), Annemarie Baltay (USGS), Bertrand Rouet-Leduc (Kyoto University) and David Kammer (ETH Zürich) to discuss how such a multi-disciplinary approach can advance our understanding of Earthquakes.

Can you give a brief overview of what your scientific work looks like and from what angle you approach Earthquakes?

**Bertrand:** My research on earthquakes is focused on the topics of earthquake nucleation and the interaction between slip modes - the way tectonic stress is released. A variety of slip modes exist, with dynamic earthquakes and creep at both ends of a spectrum that encompasses slow slip events of varied duration and scale. Many questions remain on the interplay between the members of this spectrum, including what may determine how and why a slow slip event may degenerate into an earthquake.

My research approaches these questions of earthquake nucleation and the interplay between slip modes from two angles: at multiple scales and using data science. I develop machine learning-based methods to detect seismic and geodetic signals from the scale of laboratory experiments, to the scale of subduction zones.
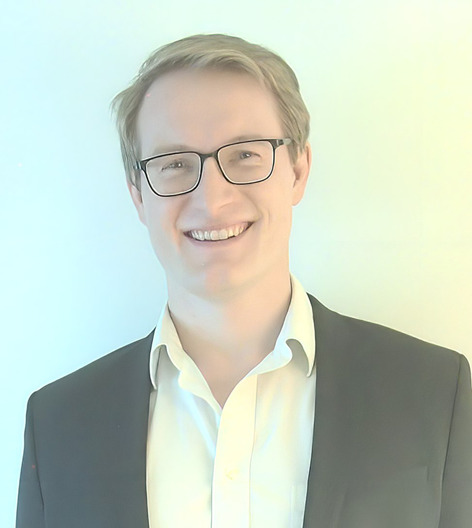


**Marie:** My research aims to understand the physics of fluid-induced earthquakes. Anthropogenic fluid injections during hydraulic fracturing, reservoir impoundment, the injection of waste water or CO2 storage can induce small stress perturbations in the underground and lead to fault reactivation and enhanced seismic activity. Moreover, long-lasting regular natural earthquake sequences are often associated with elevated pore fluid pressures at seismogenic depths. The mechanisms that govern the nucleation, propagation and recurrence of fluid-induced earthquakes are poorly constrained, and our ability to assess the seismic hazard that is associated with natural and induced events remains limited. At EPFL, we aim to improve our knowledge of fluid-induced earthquake mechanisms through multi-scale experimental approaches.

We apply cm-scale friction experiments to study the effect of fluid pressure on earthquake nucleation and propagation under crustal deformation conditions during the entire earthquake cycle. dm-scale dynamic rupture experiments are in turn applied on experimental faults to investigate the influence of fluid pressure on the nucleation and propagation of ruptures. Our analysis of post-mortem experimental faults is carried out with state-of-the-art microstructural techniques. We finally aim to calibrate the theoretical friction law with friction experiments and faulted rock microstructural observations.

**David:** In my research, we aim to establish a fundamental understanding of tectonic fault ruptures as they occur during natural earthquakes. We develop theoretical and numerical models that describe the full cycle of an earthquake, including nucleation, propagation and arrest of the fault rupture, and help us to understand the mechanisms that govern earthquakes.

We pursue our objectives along multiple research axes. First, we develop numerical methods that allow us to include more complexity into earthquake fault rupture models in order to build more realistic earthquake scenarios. Second, we calibrate our models with observations from friction experiments, as described by Marie, and use them to support the analysis of observations from large-scale laboratory earthquake experiments by giving access to quantities that are not easily measured in the experiments. Finally, we use our simulation results to develop fracture-mechanics-based theoretical models of laboratory earthquakes, which we then apply to upscale the knowledge gained from large-scale experiments to the field scale and natural earthquakes.
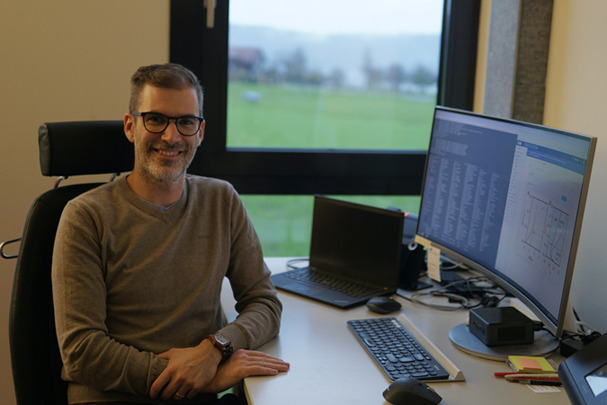


**Annemarie:** I am an observational earthquake scientist at the U.S. Geological Survey, using seismograms recorded at various distances from the earthquakes to probe what we know about both the earthquake source as well as how seismic waves propagate through Earth. I am interested in how both earthquakes and Earth control ground motions which are measured at distance, and how these reveal the earthquake source and path. I am particularly interested in earthquake stress drop, which is the amount of tectonic stress released during an earthquake rupture, and which can be estimated from the radiated seismic waves.

I further work on ground-motion models (GMMs) and their physical components and uncertainty. Reducing the latter, will ultimately lead to more precise and accurate seismic hazard maps. Currently, I am working towards physical explanations for variability in the source, site, and path components in ground motions. Ultimately, we will develop models for predicting those effects from geophysical observables, such as stress drop (for source), site velocity profiles and attenuation (for site), and whole-path attenuation (for path).
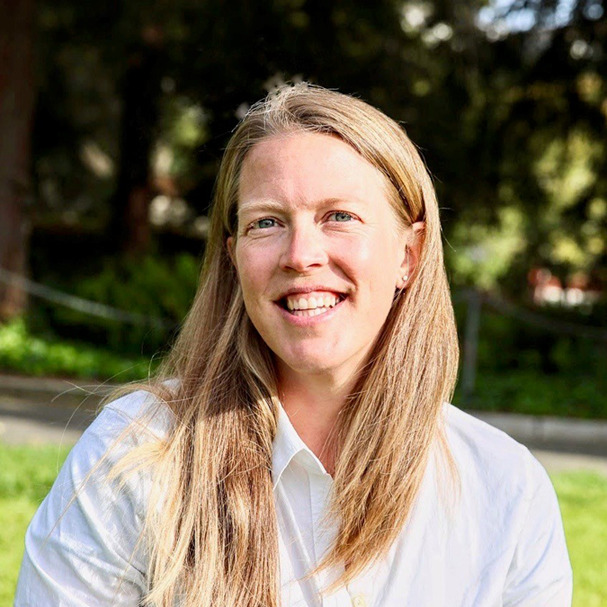


What are the most impactful recent advances in your communities and how do they add to the bigger picture in Earthquake science?

**Bertrand:** Recent physical models of the earthquake cycle and laboratory studies suggest earthquakes may nucleate during a preparatory aseismic phase of variable duration from minutes to years^1–4^. An aseismic phase is characterized through surface displacement, but the absence of notable earthquakes.

Thanks to increasing deployments of seismic and GPS stations, as well as the development of Interferometric Synthetic Aperture Radar (InSAR), the observation of such aseismic deformation is becoming common, from continuous aseismic slip^5,6^ to week-long slow slip events^7,8^. The systematic observation of deformation events on faults is getting closer and may soon give definite answers on the interaction between slip modes and on earthquake nucleation.

**Marie:** Aseismic slip plays an important role for us as well - recent laboratory and natural observations suggest it to be one of the triggering mechanisms of fluid-induced earthquakes. Whereas other trigger mechanisms do exist as well, aseismic slip has an important role insofar that it can induce seismicity in regions beyond the fluid pressurized zone and hence potentially increase the seismic hazard area. Thus, it is critical for us to not only understand the mechanisms that cause fault slip, but also the conditions that lead to (a)seismic slip.

**David**: Our community is continuously pushing the theoretical and numerical approaches to create more realistic models for the full earthquake cycle. One important contribution in the large sense is the community code verification exercises^9^, in which various numerical codes are compared and benchmarked. This is a very important contribution to continue supporting rigor and reproducibility in our field, and I believe this will have long-lasting impact.

Other recent advances that I am personally very excited about are efforts to use numerical simulations to make theoretical models, which are often very simple, a degree more realistic, but in a fundamental way. A very nice example^10,11^ is the development of theoretical models for elongated earthquake ruptures. Others include theoretical models for the propagation speed of frictional ruptures^12,13^, fluid-driven fault rupture^14,15^, and earthquake scaling^16,17^.

Finally, there are exciting efforts to enhance numerical simulations with more complexity, such as realistic fault geometry, multi-physical fault phenomena, and fault heterogeneity^18–22^.

**Annemarie**: In earthquake seismology, we are starting to explore new ways to utilize the vast amounts of available data more efficiently. Novel machine learning (ML) techniques help us to improve our earthquake catalogs, in particular to understand seismic sequences for smaller and much more frequent events. ML is further applied to mine the ambient seismic wavefield to discover tectonic tremor which helps to track plate motions or map the Earth’s interior. This includes more effectively regressing instrumental records of moderate and large earthquakes which are spatially variable, to develop so-called non-ergodic ground-motion models, with increasing sophistication and customization; and even interpreting felt earthquake reports from citizen responders to get a better idea of how people experience shaking, a topic that we are currently working on now.

What are the most pressing research questions your respective communities are working on at the moment?

**Bertrand**: Systematically observing deformation events on faults may well be key to understanding the interaction between modes of slip and earthquake nucleation, and might provide observables that may allow discriminating between a harmless slow slip event and an aseismic precursor to a major earthquake.

However, current geodetic methods cannot always resolve small (km-scale) day- to week-long events of slip, and doing so involves manual processing and analysis that cannot scale to the systematic and global observation of deformation events. Progress towards automatic detection of tectonic events, with recent successes from automatic detection of aseismic slip^23^ to earthquakes^24^, is among the most pressing research topics in the quest towards a better understanding of the spectrum of slip modes, the interaction between slip modes, and earthquake nucleation.

**Marie:** One major research task is to determine what controls the onset of dynamic instability, i.e. the competition between frictional aseimic preslip and fluid diffusion fronts. We further try to get a better handle on both what’s controlling the maximum magnitude of fluid-induced events, but also whether the maximum magnitude scales with a number of parameters (injected volume, the pre-stress, stress state, fault area, fluid injection rate, the compressibility of the fluid or a combination of these). A final question is whether heterogeneity enters into the scaling.

**David:** Physically speaking, there are many questions related to the earthquake cycle and the processes governing it. For instance, what is the exact nucleation process of an earthquake or how do natural fault ruptures arrest? Many of these questions are directly related to a need for a better understanding of fault friction properties (e.g., fracture energy) and multi-physical phenomena (e.g. pore pressure, temperature) under natural conditions, and for more information about fault heterogeneity and its effect on earthquake mechanics.

From a theoretical perspective, there is an important question on reconciling observations from small-scale rock experiments, with large-scale laboratory earthquake experiments, and field observations. Can we build models that consolidate our knowledge from the lab with observations from the field?

Are there specific research questions you would like to see addressed by another community?

**Bertrand**: As progress towards automation of tectonic deformation is becoming a pressing issue to keep progressing towards a better understanding of earthquakes, the involvement of the data science and machine learning (ML) communities could make all the difference. Similar to how developments of ML for the life sciences have become ubiquitous, developments of ML specifically for the earth sciences will hopefully become another important area of applied ML research.

**Marie:** As an experimentalist we always try to make our measurements as precise and fast as possible, as close to the fault, and on as many points as possible. Digital image correlation allows fast and precise measurements of displacement for experiments performed without confining pressure. The development of distributed fiber optic measurement has just started to produce excellent results in pressure and temperature, and we intend to deepen our collaboration with this community.

**David:** As modelers we are always relying on experimental data for calibration and validation of our models (as a return we provide the opportunity of generalizing the experimental results). For this reason, more precise experimental observations of the local constitutive friction law at realistic conditions (e.g. high rupture speed and high contact pressure) would be very helpful. This is, of course, technically very challenging, but I would like to push for more direct collaboration between experimental and theoretical researchers, as this could lead to important progress in our fundamental understanding of earthquake mechanics.

**Annemarie:** As an observational earthquake seismologist, I think we need to strengthen our link in two directions -- earthquake simulations, both dynamic and kinematic, and laboratory experiments. In both of those cases, inputs such as stress, slip, dimension or material properties can be set and controlled, parameters which we have difficulty resolving in detail or with reliability observationally. We need to continue to validate the simulations, to ensure that they are capturing the correct physics and earth properties, and on the lab side, push the scale of experiments to bridge the link to in-situ earthquakes. Of course, the collaboration between all the disciplines is essential to ensure results and interpretations are brought together.

How would you like to see the link between earthquake policy and hazard mitigation strategies strengthened in regards to your research area?

**Bertrand:** In the not so distant future, tectonic deformation may be continuously monitored using data science and ML models on both seismic and geodetic data, notably yielding improved mappings of fault locking and slip budget, with the potential to inform and improve models of seismic hazard.

**Marie:** The reliability of natural hazard estimates needs to rely heavily on the definition of a faulting model, which needs to be underpinned by realistic physical constraints such as fault geometry, friction and rupture laws.

**David:** I agree that data-driven and ML approaches have the potential to support the process of determining the seismic hazard. As nicely pointed-out by Marie, the models should be constrained by physical considerations. In addition to those already mentioned, I would also include constraints based on fault rupture processes, such as energy balance, rupture mode, and propagation/arrest conditions.

**Annemarie:** As we continually refine and update our models of seismicity rates and occurrence, we have more detailed, specific, accurate models for seismic shaking, which also results in models that are more precise and less variable. Spatial and temporal dependence on finer scales could be incorporated into hazard and forecast products; in the case of USGS products such as Operational Aftershock Forecasting, we could give communities a more accurate and precise picture of what to expect after a large earthquake, which could quell anxiety and bring better preparedness.

*This interview was conducted by Sebastian Müller.*
